# Association between triglyceride-glucose-related indices and liver-related events in patients with type 2 diabetes

**DOI:** 10.3389/fendo.2026.1760656

**Published:** 2026-04-22

**Authors:** Yanan Mi, Chenhao Ye, Deji Song, Wang Chao, Ziqi Zhang, Lei Wang, Wei Qian

**Affiliations:** 1 Institute of Digestive Diseases, Longhua Hospital, Shanghai University of Traditional Chinese Medicine, Shanghai, China; 2 School of Public Health, Shanghai University of Traditional Chinese Medicine, Shanghai, China; 3 Comprehensive Supervision and Law Enforcement Bureau, Shibei District Health Bureau, Qingdao, China; 4 Department of Hepatology, Longhua Hospital, Shanghai University of Traditional Chinese Medicine, Shanghai, China

**Keywords:** diabetic complications, liver-related events, triglyceride-glucose index, type 2 diabetes, UK Biobank

## Abstract

**Background:**

Accumulating evidence has linked the triglyceride-glucose (TyG) index and its derived measures to a broad range of diabetic complications. However, the nature of the association between these indices and subsequent liver morbidity in type 2 diabetes (T2D) patients warrants further investigation. The present analysis assessed the prospective associations between four TyG-based parameters and the incidence of liver-related events (LRE) among people with T2D.

**Methods:**

This prospective cohort study included 18,105 participants with T2D from the UK Biobank. Four TyG-related indices were assessed, including TyG, TyG-body mass index (TyG-BMI), TyG-waist circumference (TyG-WC), and TyG-waist-to-height ratio (TyG-WHtR). Cox proportional hazards models and restricted cubic spline (RCS) were used to evaluate the associations between TyG-related indices and incident LRE risk.

**Results:**

During a median follow-up of 13.4 years, 507 T2D patients developed LRE. Compared to patients in the lowest quartile, those in the highest quartiles of both TyG-WC (HR = 1.63, 95% CI 1.12-2.38) and TyG-WHtR (HR = 1.98, 95% CI 1.36-2.89) were associated with increased risk of LRE. Restricted cubic spline models confirmed linear relationships for both TyG-WC and TyG-WHtR with LRE risk. No significant associations of TyG and TyG-BMI with LRE risk were observed. Subgroup analyses demonstrated that the associations between TyG-WC/TyG-WHtR and LRE were more pronounced in high-risk populations, including excessive alcohol consumers and individuals with FIB-4 scores ≥1.3 (indicating higher liver fibrosis risk). For instance, the association between TyG-WHtR and LRE was stronger in individuals with FIB-4 ≥1.3 (HR = 2.59, 95% CI 1.65-4.07) compared to those with FIB-4 <1.3 (HR = 1.58, 95% CI 0.76-3.29).

**Conclusion:**

Among T2D patients, TyG-WC and TyG-WHtR were consistently associated with increased risk of LRE, confirming the independent clinical utility of these indices. These findings demonstrate that both indices may serve as efficient, cost-effective, and practical tools for enhancing the identification and stratification of LRE risk in clinical practice.

## Introduction

It is well-established that chronic liver disease accounts for a substantial proportion of deaths worldwide (1). A major challenge lies in the fact that most patients with advanced disease remain undiagnosed until they develop clinically overt liver-related events (LRE), including cirrhosis, decompensation, or hepatocellular carcinoma (HCC) - stages at which prognosis becomes invariably poor (2, 3). This clinical reality underscores the imperative for reliable methods to identify high-risk individuals at earlier, more treatable stages.

Epidemiological studies have consistently established type 2 diabetes (T2D) as a key determinant of LRE risk (4–6). In recognition of this association, current clinical guidelines classify T2D patients as a high-risk population warranting routine LRE surveillance, regardless of baseline liver status (7, 8). While several risk prediction tools exist for LRE monitoring (9), the Fibrosis-4 (FIB-4) score has emerged as the most widely recommended stratification tool for T2D populations by incorporating age, platelet count, and liver enzymes (4–6). However, as FIB-4 was originally developed in cohorts with established liver disease, its application to T2D patients reveals significant limitations: (1) reduced prediction accuracy compared to its performance in populations with liver diseases (7, 8), and (2) frequent assignment of patients to indeterminate-risk categories (FIB-4 1.3-2.67), which often require additional evaluations (10). These limitations emphasize the urgent need to optimize the risk identification strategy for LRE in patients with T2D.

Recent investigations have highlighted the potential of the triglyceride-glucose (TyG) index and its variants (e.g., TyG-WC, TyG-WHtR) as promising biomarkers, demonstrating strong correlations with hepatic injury, inflammation, and fibrosis progression (11–14). These indices capture underlying insulin resistance - a fundamental pathophysiological mechanism in liver disease progression - and may provide additional prognostic information compared to conventional markers (15, 16). Currently, their prognostic utility for incident LRE in T2D populations remains unestablished. Drawing upon the prospective UK Biobank cohort, this research sought to assess the relevance of four TyG-related parameters to the risk of incident LRE among individuals with T2D.

## Methods

### Study design and individuals

**Figure 1:**
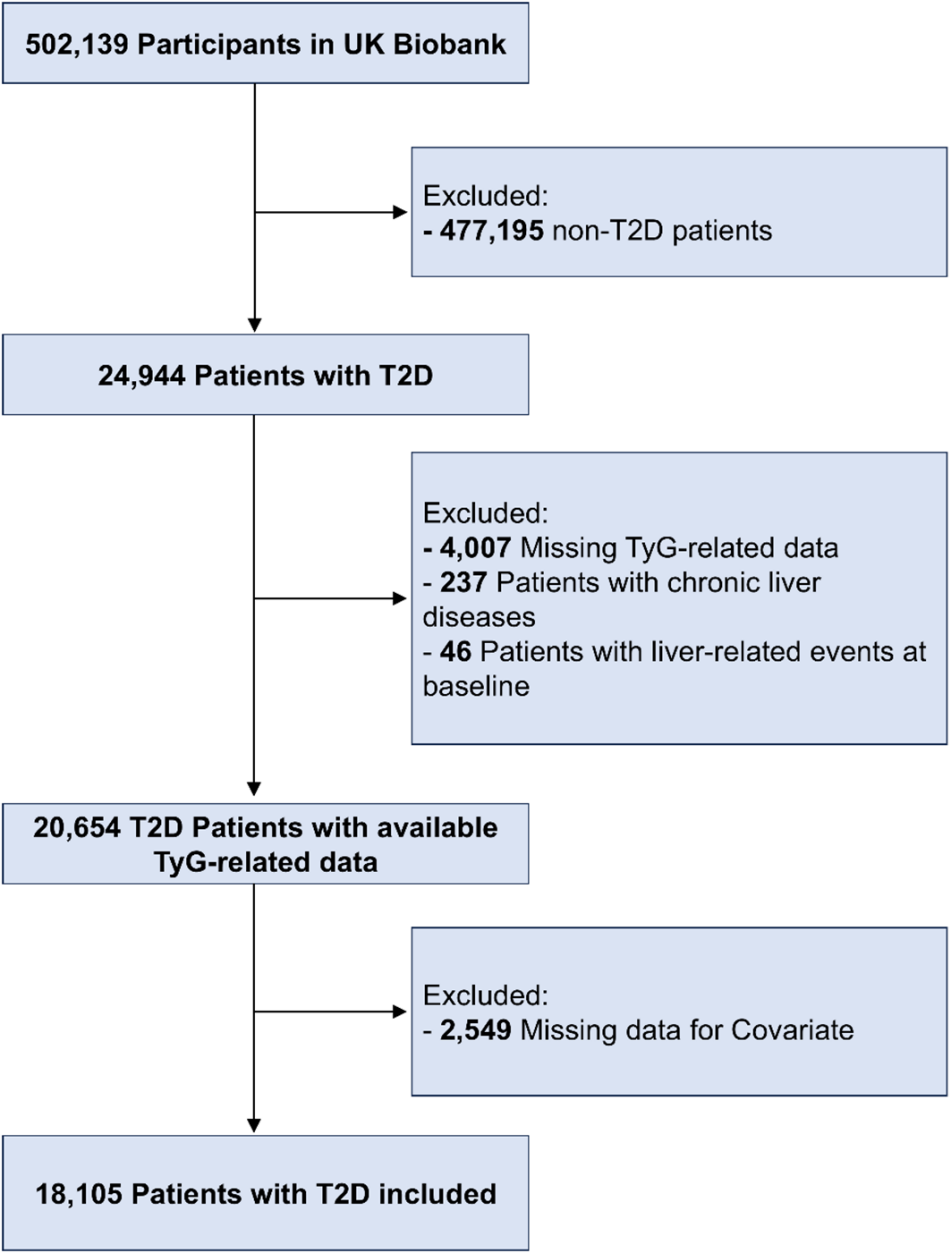
Flowchart of study population selection.

Between 2006 and 2010, the UK Biobank initiative recruited more than 500,000 participants. Baseline visit collected comprehensive data covering demographics, lifestyle factors, health status, and biological samples. Conditional on ethical approval from the Northwest Multi-Centre Research Ethics Committee, the UK Biobank study required written informed consent from every participant prior to enrollment. Of the 502,139 participants in the cohort, we initially identified 24,944 participants with prevalent T2D through an algorithm incorporating diagnostic records, diabetes classification, medication records, and complication data (17). We applied the following sequential exclusion criteria: missing data on TyG-related indices (n=4,007), a history of chronic liver disease (n=237, including viral, alcoholic, or autoimmune hepatitis), a baseline diagnosis of LRE (n=46), or missing covariate data (n=2,549). Following all exclusions, we established an analytical cohort of 18,105 individuals with T2D (**Figure 1**).

### Calculation of TyG-related indices

As part of the baseline assessment, peripheral blood was drawn from every participant for subsequent biochemical and biomarker analysis. From participants with obtained consent, blood samples were separated in components at -80 °C, with subsequent biochemical measurements performed on a Beckman Coulter AU5800. Via the UKB website, full methodological and quality control documentation is accessible. Following established methodology, the core TyG index was first derived as Ln [triglycerides (mg/dL) × fasting glucose (mg/dL)/2]. Three additional parameters were then derived from the product of TyG and respective anthropometric measures: TyG-BMI (TyG × BMI), TyG-WC (TyG × waist circumference in cm), and TyG-WHtR (TyG × waist-to-height ratio) (18). Fasting glucose and triglyceride concentrations were converted from mmol/L to mg/dL by multiplying by 18 and 88.57, respectively.

### Assessment of outcomes

A composite endpoint of incident LRE served as the primary outcome measure, defined as a diagnosis of cirrhosis (ICD-10 code K74), hepatic decompensation (ICD-10 codes K72, K76, I85, I86), HCC (ICD-10 code C22), liver transplantation (ICD-10 code Z944; OPCS4 code J01), or liver-related mortality (ICD-10 codes K72, K74, K76, I85, I86.4 and C22), identified using specific ICD-10 and OPCS-4 codes. Follow-up duration was calculated from the date of enrollment to the date of whichever came first: (1) the occurrence of LRE, (2) death, or (3) administrative censoring at the end of the observation period (until 2022.10.31).

### Covariates

Covariates were included according to predefined criteria derived from prior evidence of their relevance. The following covariates were adjusted for in the analyses: age; sex (male/female); body mass index (BMI, continuous); smoking status (never, former or current); ethnicity (White/non-White); educational attainment (below high school/high school/college and above); the Townsend Deprivation Index (TDI, continuous); excessive alcohol consumption (yes/no); HbA1c (continuous, %); hypertension (yes/no); statin use (yes/no); and the FIB-4 index (at <1.3/at ≥1.3); excessive alcohol consumption (yes/no). Hypertension was ascertained based on meeting any criterion from the following: systolic/diastolic blood pressure ≥140/90 mmHg; treated hypertension, ascertained by either clinical diagnosis, active pharmacotherapy or a relevant ICD-10 code. The FIB-4 index was obtained by applying the established formula, and values <1.3 were considered indicative of low risk of advanced fibrosis according to standard thresholds (19).

### Statistical analysis

Continuous variables were presented as mean (standard deviation) and categorical variables are presented as frequency (percentage). The cumulative incidence of LRE was estimated using the Kaplan-Meier method. We used restricted cubic splines with three knots to explore potential non-linear relationships. Hazard ratios (HRs) and their 95% Confidence Intervals (CIs) for the associations between TyG-related indices and LRE risk were calculated using Cox proportional hazard models. The multivariable model adjusted for the following covariates based on prior knowledge: age, sex, ethnicity, educational level, TDI, BMI, smoking status, drinking status, HbA1c, use of statins, hypertension, and FIB-4. Multicollinearity among covariates included in the Cox regression model was assessed using variance inflation factors (VIFs), with a VIF > 5 indicating potential multicollinearity. The proportional hazards assumption was tested using Schoenfeld residuals, and no violation was observed (**Supplementary Table 1**). We further performed subgroup analyses by FIB-4 (<1.3/≥1.3), age (<60 years/≥60 years), ethnicity (White/non-White), smoking (yes/no), BMI (<30 kg/m^2^/≥30 kg/m^2^), sex (male/female) and excessive alcohol consumption (yes/no). The BMI cutoff of 30 kg/m² was chosen because it represents the standard World Health Organization threshold for obesity (18, 20). To evaluate potential interaction effects, we incorporated product terms between the TyG-related parameters and stratifying factors into the models, with their statistical significance assessed using Wald tests. For subgroup analyses, multiple comparisons were adjusted using the false discovery rate (FDR) method according to the Benjamini–Hochberg procedure. The predictive performance of the TyG-related indices for LRE was assessed using time-dependent receiver operating characteristic (tROC) curve analysis, and the time-dependent area under the curve (tAUC) was calculated. The stability of the primary associations was examined via a set of sensitivity analyses; specifically, to mitigate reverse causation, analyses were repeated after excluding individuals diagnosed with an LRE within the first year of follow-up. Secondly, we further adjusted for C-reactive protein levels in the multivariable models to evaluate whether the observed associations were independent of systemic inflammation. Thirdly, dietary patterns were further adjusted for in the multivariable models using an alternative Mediterranean diet score, which was assessed according to previously established methods (21). Fourthly, to address missingness in covariates, multiple imputations were utilized; all statistical analyses were then repeated using these imputed datasets. Fifthly, we evaluated the associations between TyG−related indices and each component of liver−related events, including cirrhosis, HCC, and liver−related mortality. Sixthly, we accounted for competing risks by considering non-liver-related mortality as a competing event and estimated subdistribution hazard ratios (sHR) for the associations between TyG-related indices and LRE using Fine-Gray competing risk regression models. Finally, because collinearity was observed between TyG-BMI and BMI, we reported the results from a multivariable model that did not include BMI to avoid potential multicollinearity. Statistical analyses employed R software (V4.2.2). Statistical significance was defined as a two-sided p-value < 0.05.

## Results

This study enrolled 18,105 T2D patients with an average age of 60.12 ± 6.86 years, comprising 36.6% females and 63.4% males. The baseline mean values for TyG index, TyG-WHtR, TyG-WC, and TyG-BMI were 9.24 ± 0.69, 292.51 ± 61.61, 958.13 ± 162.14, and 5.67 ± 0.94, respectively. Compared with the non-LRE group, participants who developed LRE were older, more likely to be male, White, and less educated. They also had higher rates of obesity, smoking history, and excessive alcohol consumption. Clinically, the LRE group exhibited more frequent abnormalities in liver enzymes and glucose-lipid metabolism, along with elevated FIB-4 scores (**Table 1**).

**Table 1:** Baseline characteristics of participants.

Characteristic	Overall N = 18,105	Non-LRE N = 17,598	LRE N = 507	P value
Age (years)	60.12 (6.86)	60.10 (6.87)	60.94 (6.38)	0.013
Female	6,631 (36.6%)	6,490 (36.9%)	141 (27.8%)	<0.001
White	15,492 (88.1%)	15,464 (87.9%)	478 (94.3%)	<0.001
College or above	9,164 (50.6%)	8,945 (50.8%)	219 (43.2%)	<0.001
TDI	-0.45 (3.41)	-0.46 (3.41)	-0.21 (3.44)	0.097
BMI (kg/m^2^)	31.57 (5.85)	31.53 (5.84)	33.16 (5.93)	<0.001
Waist Circumference (cm)	103.42 (14.10)	103.27 (14.07)	108.89 (14.00)	<0.001
Smoking status				<0.001
Never	8,041 (44.4%)	7,872 (44.7%)	169 (33.3%)	
Former	8,065 (44.5%)	7,789 (44.3%)	276 (54.4%)	
Current	1,999 (11.0%)	1,937 (11.0%)	62 (12.2%)	
Excessive alcohol consumption	3,295 (18.2%)	3,156 (17.9%)	139 (27.4%)	<0.001
Hypertension	15,198 (83.9%)	14,751 (83.8%)	447 (88.2%)	0.009
CVD	2,201 (12.2%)	2,117 (12.0%)	84 (16.6%)	0.002
CKD	846 (4.7%)	814 (4.6%)	32 (6.3%)	0.076
Use of Statins	12,540 (69.3%)	12,194 (69.3%)	346 (68.2%)	0.614
ALT (U/L)	29.22 (17.22)	28.85 (16.56)	42.02 (30.00)	<0.001
AST (U/L)	27.97 (12.68)	27.52 (11.80)	43.58 (25.67)	<0.001
GGT (U/L)	51.78 (59.82)	49.14 (51.82)	144.70 (161.67)	<0.001
Glucose (mmol/L)	7.42 (3.20)	7.40 (3.18)	7.99 (3.64)	<0.001
HbA1c (%)	6.91 (1.24)	6.90 (1.24)	7.00 (1.35)	0.219
TG (mmol/L)	2.15 (1.21)	2.15 (1.21)	2.20 (1.25)	0.452
TC (mmol/L)	4.49 (1.02)	4.49 (1.02)	4.33 (1.07)	<0.001
HDL-C (mmol/L)	1.18 (0.31)	1.18 (0.31)	1.15 (0.32)	0.005
LDL-C (mmol/L)	2.70 (0.76)	2.70 (0.76)	2.59 (0.77)	<0.001
FIB-4				<0.001
<1.3	9,479.0 (52.4%)	9,358.0 (53.2%)	121.0 (23.9%)	
1.3-2.67	7,911.0 (43.7%)	7,652.0 (43.5%)	259.0 (51.1%)	
>2.67	715.0 (3.9%)	588.0 (3.3%)	127.0 (25.0%)	
TyG	9.24 (0.69)	9.24 (0.69)	9.33 (0.71)	0.007
TyG-BMI	292.51 (61.61)	292.01 (61.51)	309.95 (62.79)	<0.001
TyG-WC	958.13 (162.14)	956.41 (161.83)	1,017.67 (161.81)	<0.001
TyG-WHtR	5.67 (0.94)	5.66 (0.93)	5.99 (0.94)	<0.001

TDI, Townsend Deprivation Index; CVD, Cardiovascular Disease; CKD, Chronic Kidney Disease; ALT, Alanine Aminotransferase; AST, Aspartate Aminotransferase; GGT, Gamma-Glutamyl Transferase; HbA1c, Hemoglobin A1c; TG, Triglycerides; TC, Total Cholesterol; HDL-C, High-Density Lipoprotein Cholesterol; LDL-C, Low-Density Lipoprotein Cholesterol; FIB-4, Fibrosis-4 Score; TyG, Triglyceride-Glucose Index; TyG-BMI, Triglyceride-Glucose-Body Mass Index; TyG-WC, Triglyceride-Glucose-Waist Circumference; TyG-WHtR, Triglyceride-Glucose-Waist-to-Height Ratio.

**Figure 2:**
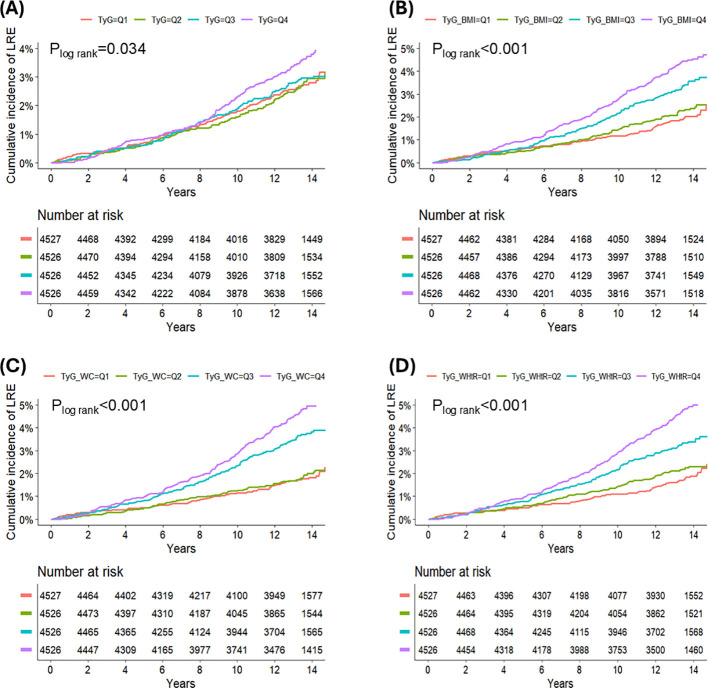
Cumulative incidence of liver-related events in patients with type 2 diabetes stratified by TyG-related index quartiles. **(A)** TyG index Kaplan–Meier curve, **(B)** TyG–BMI Kaplan–Meier curve, **(C)** TyG–WC Kaplan–Meier curve, **(D)** TyG–WHtR Kaplan–Meier curve.

**Figure 3:**
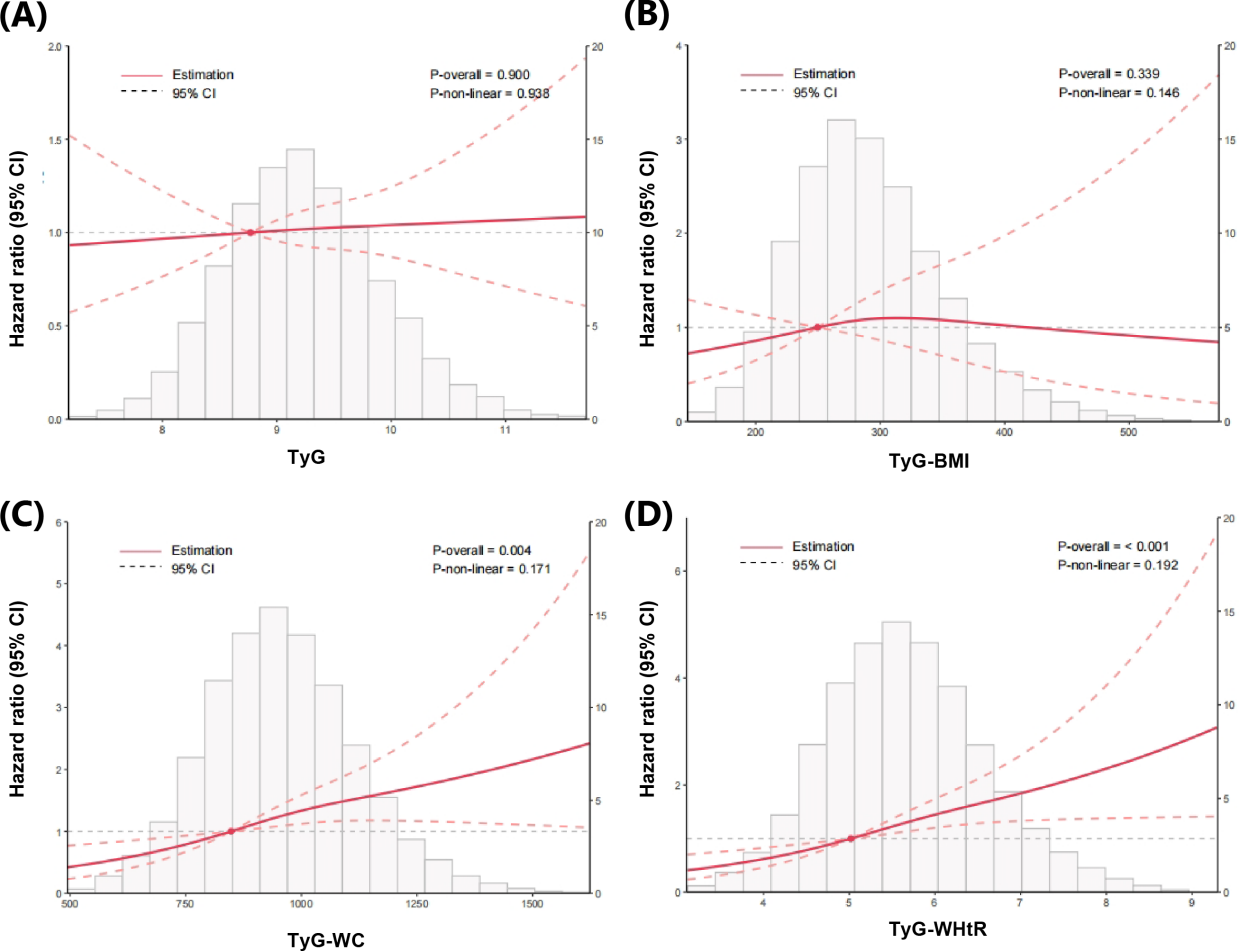
The association between TyG-related indices and liver-related events in patients with type 2 diabetes evaluated by restricted cubic splines. Models were adjusted for age, sex, ethnicity, Townsend deprivation index, educational attainment, body mass index, smoking status, excessive alcohol consumption, HbA1c, hypertension, use of statins, and FIB-4. Dashed lines indicate 95% confidence intervals. **(A)** TyG index, **(B)** TyG–BMI index, **(C)** TyG–WC index, **(D)** TyG–WHtR index.

Over a median of 13.4 years (IQR, 12.5–14.2) of follow-up, 507 LRE were documented. The top quartile (Q4) of TyG indices was correlated with the greatest cumulative incidence of LRE, as shown in **Figure 2** (*P*_logrank_ < 0.05). Analyses using restricted cubic splines revealed consistently linear relationships for all TyG-related indices with respect to LRE risk (**Figure 3**; all nonlinear *P* > 0.05). After multivariable adjustment, compared with the reference group (the lowest quartile, Q1), the third quartile (Q3) and fourth quartile (Q4) of TyG-WC revealed a significant association indicative of higher risk of LRE (Q3: HR = 1.57 [1.15, 2.15], *P* = 0.005; Q4: HR = 1.63 [1.12, 2.38], *P* = 0.012). Similarly, the Q3 and Q4 of TyG-WHtR also showed significant positive associations with LRE risk (Q3: HR = 1.59 [1.17, 2.18], *P* = 0.004; Q4: HR = 1.98 [1.36, 2.89], *P* < 0.001) (**Table 2**). In contrast to TyG-WC and TyG-WHtR, fully adjusted analyses identified no significant link between LRE risk and either the TyG index or TyG-BMI. Likelihood ratio tests yielded results similar to those of the primary analyses (**Supplementary Table 2**). The tROC curve analyses showed that the 10-year tAUC for predicting LRE was 0.53 (0.50-0.57) for TyG, 0.61 (0.57-0.64) for TyG-BMI, 0.62 (0.59-0.65) for TyG-WC, and 0.62 (0.59-0.65) for TyG-WHtR (**Supplementary Figure 1**). The optimal cut-off values determined by the Youden index were 9.91 for TyG, 317.22 for TyG-BMI, 977.40 for TyG-WC, and 5.66 for TyG-WHtR.

**Figure 4:**
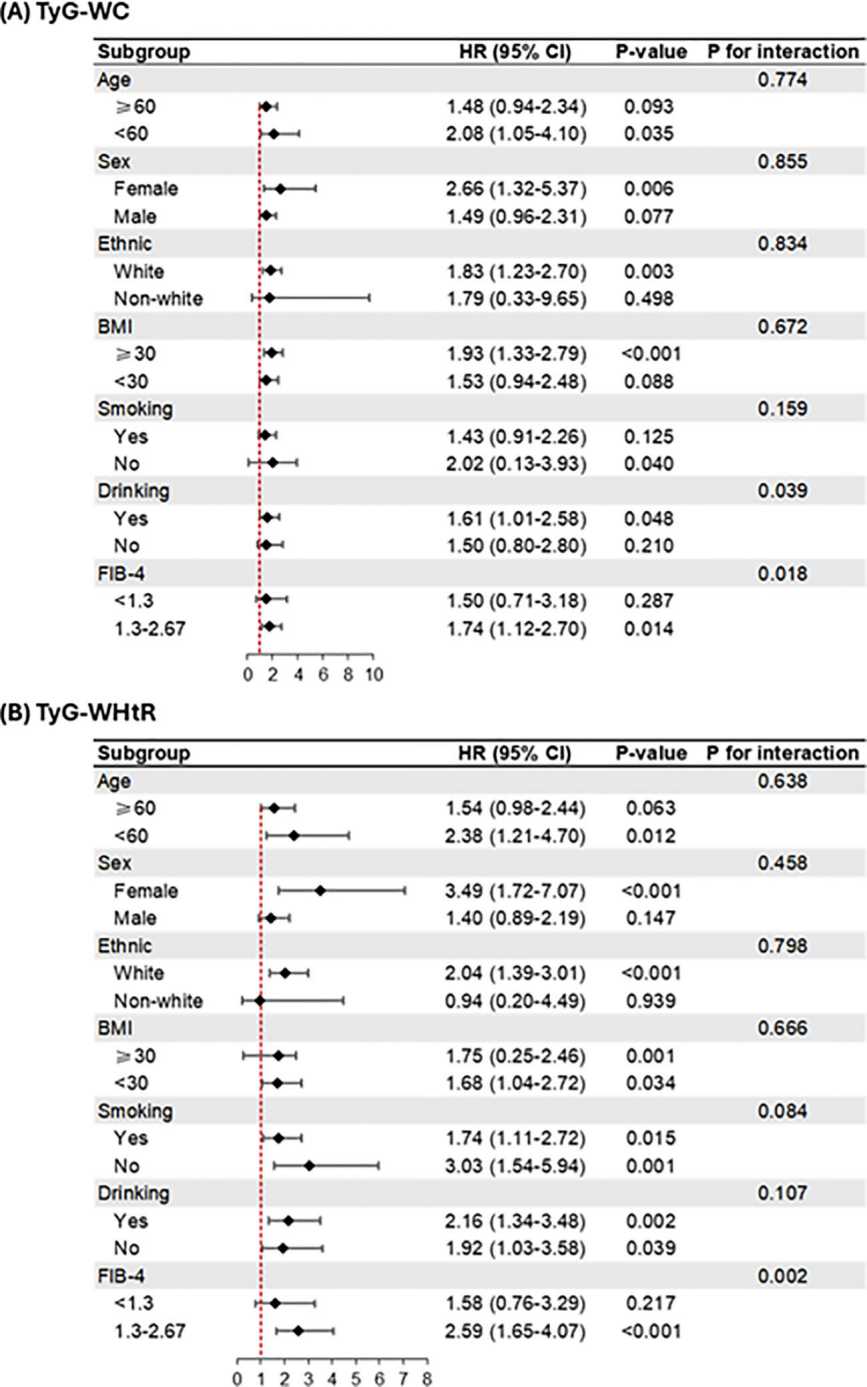
Subgroup analyses the relationships between TyG-related indices and liver-related events in patients with type 2 diabetes. Models were adjusted for age, sex, ethnicity, Townsend deprivation index, educational attainment, body mass index, smoking status, excessive alcohol consumption, HbA1c, hypertension, use of statins, and FIB-4.

The interaction term test confirmed a significant interaction of these indices with the FIB-4 score. (**Figure 4**, *P* for interaction < 0.05). The relationship between TyG-WC and TyG-WHtR with LRE risk was more pronounced among individuals with a FIB-4 score ≥ 1.3 (TyG-WC, HR = 1.74 [1.12, 2.70]; TyG-WHtR, HR = 2.59 [1.65, 4.07]) compared to those with FIB-4 <1.3 (TyG-WC, HR = 1.50 [0.71, 3.18]; TyG-WHtR, HR = 1.58 [0.76-3.29]). Similarly, a considerable interaction was found between TyG-WC and excessive alcohol consumption (**Figure 4**). The association of TyG-WC with LRE was greater in individuals with excessive alcohol intake (HR = 1.61 [1.01, 2.58]) compared to those without (HR = 1.50 [0.80, 2.80]). The primary findings remained robust across all sensitivity analyses. Specifically, after excluding early incident cases within one year of follow-up, both TyG-WC and TyG-WHtR remained pronouncedly associated with LRE risk (**Supplementary Table 3**). Although additional adjustment for C-reactive protein levels resulted in modest attenuation of effect estimates, the associations remained significant (**Supplementary Table 4**). In a subgroup of participants with available dietary data, only TyG−WHtR was significantly associated with LRE, and this association remained robust after further adjustment for dietary patterns (**Supplementary Table 5**). The results were further confirmed in analyses incorporating multiple imputations for missing covariates and in competing risk models accounting for competing events (**Supplementary Tables 6**, **7**). For individual LRE components, TyG−WC was consistently associated with all outcomes, whereas TyG−WHtR showed no significant association with HCC (**Supplementary Table 8**). In the multivariable model excluding BMI, the associations of TyG−WC and TyG−WHtR with LRE were strengthened, and a significant association between TyG−BMI and LRE was also observed (**Supplementary Table 9**). The consistent associations observed across different sensitivity analyses support the potential utility of TyG-WC and TyG-WHtR as consistent and clinically useful indicators for LRE risk stratification in T2D.

**Table 2:** Associations between TyG-related indices and risk of incident liver-related events in patients with type 2 diabetes.

Characteristic	Event/ Number	Model 1		Model 2	
		HR (95% CI)	*P* value	HR (95% CI)	*P* value
TyG					
Q1	118/4527	Ref		Ref	
Q2	115/4526	0.97 (0.75, 1.26)	0.822	0.91 (0.70, 1.18)	0.482
Q3	120/4526	1.03 (0.80, 1.32)	0.841	0.92 (0.71, 1.19)	0.526
Q4	154/4526	1.32 (1.04, 1.68)	0.022	0.98 (0.75, 1.28)	0.878
*P* for trend			0.021		0.901
TyG-BMI					
Q1	86/4527	Ref		Ref	
Q2	96/4526	1.13 (0.84, 1.51)	0.426	1.00 (0.74, 1.37)	0.989
Q3	144/4526	1.69 (1.29, 2.21)	<0.001	1.31 (0.94, 1.81)	0.111
Q4	181/4526	2.17 (1.68, 2.81)	<0.001	1.44 (0.92, 2.24)	0.106
*P* for trend			<0.001		<0.001
TyG-WC					
Q1	79/4527	Ref		Ref	
Q2	80/4526	1.02(0.75, 1.39)	0.897	0.90(0.65, 1.24)	0.509
Q3	154/4526	1.99(1.52, 2.62)	<0.001	1.57(1.15, 2.15)	0.005
Q4	194/4526	2.61(2.01, 3.39)	<0.001	1.63(1.12, 2.38)	0.012
*P* for trend			<0.001		<0.001
TyG-WHtR					
Q1	80/4527	Ref		Ref	
Q2	93/4526	1.17(0.86, 1.57)	0.315	1.11(0.81, 1.51)	0.514
Q3	140/4526	1.79(1.36, 2.35)	<0.001	1.59(1.17, 2.18)	0.004
Q4	194/4526	2.55(1.97, 3.31)	<0.001	1.98(1.36, 2.89)	<0.001
*P* for trend			<0.001		<0.001

Model 1: unadjusted for covariates.

Model 2: adjusted for age, sex, ethnicity (White and non-white), education (below high school level, high school and college or above), Townsend Deprivation Index, body mass index, smoking status (never, former or current), excessive alcohol consumption (yes or no), HbA1c, hypertension (yes or no), use of statins (yes or no), FIB-4 (1.3< or ≥1.3).

## Discussion

In this large-scale prospective cohort study based on the UK Biobank, we demonstrated that higher levels of TyG-WC and TyG-WHtR were associated with an increased risk of incident LRE in individuals with T2D. Notably, these associations were more pronounced among those with excessive alcohol consumption or higher FIB-4 scores. These findings position TyG-WC and TyG-WHtR as practical, low-cost tools for identifying T2D patients at heightened risk of LRE, particularly in subgroups with established risk factors. The results underscored the clinical utility of integrating these indices into routine risk stratification to enable early identification of high-risk populations.

TyG and its derived indices have garnered significant attention as established predictors of both fatal and non-fatal cardiovascular outcomes in patients with T2D (22–26). For instance, these indices have shown predictive capacity for cardiovascular outcomes such as myocardial infarction, revascularization, and cardiovascular autonomic neuropathy (22–25), which emphasize the prognostic relevance of TyG-based markers for cardiovascular outcomes among individuals with T2D. Accumulating data have further implicated TyG-derived indices in the spectrum of liver pathologies, from MASLD and metabolic dysfunction-associated steatohepatitis (MASH) to fibrosis (14, 27, 28), each a recognized risk factor for progressive LRE. Consistent with our findings, a recent cross-sectional study based on the National Health and Nutrition Examination Survey (NHANES) cohort also confirmed that TyG-derived indices are significantly associated with advanced liver fibrosis in patients with T2D, further supporting the value of these indices for liver disease progression in diabetic populations (29). However, prospective evidence regarding their value as direct predictors of LRE remains limited. Our study provides the first prospective evidence linking TyG-WC and TyG-WHtR to incident LRE among T2D patients. Specifically, compared to the first quartile, patients in the top quartile of TyG-WC and TyG-WHtR had a 63% and 98% increased risk of LRE, respectively. These associations proved robust to extensive covariate adjustment and sensitivity analyses. However, we observed no statistically significant link between the original TyG index and LRE risk.

C-reactive protein (CRP) is a well-established marker of systemic inflammation. However, the associations between TyG-related indices and LRE risk remained robust after additional adjustment for CRP, suggesting that these indices capture pathogenic mechanisms beyond systemic inflammation. Mechanically, the superior performance of TyG-WC and TyG-WHtR compared with TyG alone in predicting incident LRE may reflect the synergistic effects of systemic insulin resistance (IR) and abdominal adiposity on liver disease progression. The TyG index is a well−established surrogate marker of IR. IR accelerates lipolysis in peripheral adipose tissue, resulting in an increased flux of free fatty acids to the liver, which constitutes a major source of hepatic lipid accumulation. In parallel, anthropometric measures such as waist circumference and waist−to−height ratio reflect greater visceral adiposity and central fat storage, which may further amplify this pathological process. The ensuing hepatic lipid overload promotes lipotoxicity, mitochondrial dysfunction, and oxidative stress, accompanied by the activation of Kupffer cells and hepatic stellate cells. These processes collectively drive hepatic inflammation and fibrogenesis, which increase the risk of cirrhosis, and adverse liver-related outcomes. Therefore, combining TyG with anthropometric indices may more accurately capture the complex pathophysiological mechanisms underlying liver disease progression and provide improved risk stratification for adverse liver outcomes.

Currently, in light of the established link between T2D and accelerated fibrosis progression, clinical guidelines from several societies now recommend systematic screening with non-invasive scores to identify individuals at high risk for advanced fibrosis and subsequent LRE (4–6). While FIB-4 serves as the current first choice, its suboptimal accuracy in diabetic populations may lead to potential under detection (7, 8, 30). Our research documented that TyG-WC and TyG-WHtR can further identify individuals with increased LRE risk among the subgroup with FIB-4 ≥1.3, thereby optimizing risk stratification. By utilizing readily available clinical measures, these indices offer a scalable strategy to enable timely screening of at-risk individuals, which is especially valuable in routine care or resource-limited environments.

Notable strengths of our study comprise: (1) leveraging an extensive, comprehensively phenotypic T2D cohort from the UK Biobank, providing substantial power for primary and stratified analyses; (2) extensively adjusted for potential confounders encompassing demographic, lifestyle, metabolic, and clinical factors; and (3) demonstrating consistent results across several sensitivity analyses, further strengthening the reliability of our conclusions. Our findings should be understood within the confines of some main limitations. Firstly, TyG-related indices were assessed only at baseline, and repeated measurements were not available for most participants. Given that metabolic status and anthropometric measures may change over time, a single baseline assessment may not fully capture long-term exposure. Future studies with repeated measurements are therefore warranted to evaluate the longitudinal trajectories of TyG-related indices and to better assess their value as dynamic risk stratification and follow-up tools. Secondly, although we adjusted for a wide range of covariates, unmeasured factors—such as the severity of hepatic steatosis—may still have influenced the observed associations. Moreover, given the observational design of the study, causal inferences cannot be established. Thirdly, after correction for multiple testing, the associations in some subgroups became non−significant, which may be partly attributable to the limited sample sizes within these subgroups. Therefore, the subgroup findings should be interpreted with caution. Fourthly, the predictive performance of the TyG-related indices was only moderate. Therefore, it should not be considered a high-precision predictive tool when used alone. Instead, it may be more suitable as a screening indicator with relatively high sensitivity, or for sequential use in combination with existing risk stratification tools. Lastly, since our cohort primarily consisted of White individuals, the generalizability of these findings to other ethnic groups may be limited. Future studies in more ethnically diverse populations are needed to validate our results.

## Conclusion

In conclusion, our findings demonstrate that TyG-WC and TyG-WHtR are associated with a higher risk of LRE in patients with T2D. This association was particularly pronounced among individuals with excessive alcohol consumption or a FIB-4 score ≥1.3. These outcomes demonstrated that TyG-WC and TyG-WHtR may constitute accessible and low-cost tools to improve risk stratification for LRE, facilitating earlier intervention in clinical practice and thus helping to reduce the associated clinical burden.

## Data Availability

The raw data supporting the conclusions of this article will be made available by the authors, without undue reservation. This study was conducted using data from the UK Biobank (Application Number: 221713).
