# Supplementary material for: Association between triglyceride-glucose-related indices and liver-related events in patients with type 2 diabetes
Source: Front Endocrinol (Lausanne). 2026 Apr 22;17:1760656. doi: 10.3389/fendo.2026.1760656 (PMC13143714; doi:10.3389/fendo.2026.1760656)
Supplement: Supplementary file 1 [file Table1.docx]

**Supplementary Table 1. Schoenfeld residual tests for the proportional hazards assumption in Cox models of TyG-related indices**

| **Model A: TyG** | | **Model B: TyG-BMI** | | **Model C: TyG-WC** | | **Model D: TyG-WHtR** | |
| --- | --- | --- | --- | --- | --- | --- | --- |
| **Variable** | **P value** | **Variable** | **P value** | **Variable** | **P value** | **Variable** | **P value** |
| TyG | 0.354 | TyG-BMI | 0.962 | TyG-WC | 0.564 | TyG-WHtR | 0.298 |
| Age | 0.709 | Age | 0.711 | Age | 0.725 | Age | 0.719 |
| Sex | 0.568 | Sex | 0.565 | Sex | 0.563 | Sex | 0.565 |
| Ethnicity | 0.262 | Ethnicity | 0.259 | Ethnicity | 0.259 | Ethnicity | 0.258 |
| Education | 0.405 | Education | 0.407 | Education | 0.406 | Education | 0.408 |
| Townsend Deprivation Index | 0.081 | Townsend Deprivation Index | 0.079 | Townsend Deprivation Index | 0.079 | Townsend Deprivation Index | 0.078 |
| BMI | 0.745 | BMI | 0.726 | BMI | 0.712 | BMI | 0.715 |
| Smoking | 0.828 | Smoking | 0.831 | Smoking | 0.830 | Smoking | 0.830 |
| Alcohol consumption | 0.621 | Alcohol consumption | 0.619 | Alcohol consumption | 0.623 | Alcohol consumption | 0.618 |
| HbA1c | 0.388 | HbA1c | 0.391 | HbA1c | 0.391 | HbA1c | 0.388 |
| Hypertension | 0.196 | Hypertension | 0.196 | Hypertension | 0.197 | Hypertension | 0.196 |
| Use of statins | 0.465 | Use of statins | 0.461 | Use of statins | 0.463 | Use of statins | 0.464 |
| FIB-4 | 0.502 | FIB-4 | 0.501 | FIB-4 | 0.502 | FIB-4 | 0.503 |
| **GLOBAL** | **0.605** | **GLOBAL** | **0.760** | **GLOBAL** | **0.454** | **GLOBAL** | **0.413** |

**Supplementary Table 2. Associations between TyG-related indices and liver-related events in patients with type 2 diabetes using Cox proportional hazards models with likelihood ratio tests**

| **Characteristic** | **Event/ Number** | **Model 1** | | **Model 2** | |
| --- | --- | --- | --- | --- | --- |
|  |  | **HR (95% CI)** | ***P* value** | **HR (95% CI)** | ***P* value** |
| TyG |  |  |  |  |  |
| Q1 | 118/4527 | Ref |  | Ref |  |
| Q2 | 115/4526 | 0.97 (0.75, 1.26) | 0.822 | 0.91 (0.70, 1.18) | 0.482 |
| Q3 | 120/4526 | 1.03 (0.80, 1.32) | 0.841 | 0.92 (0.71, 1.19) | 0.526 |
| Q4 | 154/4526 | 1.32 (1.04, 1.68) | 0.022 | 0.98 (0.75, 1.28) | 0.878 |
| *P* for trend |  |  | 0.021 |  | 0.901 |
| *P* for LRT |  |  | 0.040 |  | 0.830 |
| TyG-BMI |  |  |  |  |  |
| Q1 | 86/4527 | Ref |  | Ref |  |
| Q2 | 96/4526 | 1.13 (0.84, 1.51) | 0.426 | 1.00 (0.74, 1.37) | 0.989 |
| Q3 | 144/4526 | 1.69 (1.29, 2.21) | <0.001 | 1.31 (0.94, 1.81) | 0.111 |
| Q4 | 181/4526 | 2.17 (1.68, 2.81) | <0.001 | 1.44 (0.92, 2.24) | 0.106 |
| *P* for trend |  |  | <0.001 |  | <0.001 |
| *P* for LRT |  |  | <0.001 |  | 0.180 |
| TyG-WC |  |  |  |  |  |
| Q1 | 79/4527 | Ref |  | Ref |  |
| Q2 | 80/4526 | 1.02(0.75, 1.39) | 0.897 | 0.90(0.65, 1.24) | 0.509 |
| Q3 | 154/4526 | 1.99(1.52, 2.62) | <0.001 | 1.57(1.15, 2.15) | 0.005 |
| Q4 | 194/4526 | 2.61(2.01, 3.39) | <0.001 | 1.63(1.12, 2.38) | 0.012 |
| *P* for trend |  |  | <0.001 |  | <0.001 |
| *P* for LRT |  |  | <0.001 |  | <0.001 |
| TyG-WHtR |  |  |  |  |  |
| Q1 | 80/4527 | Ref |  | Ref |  |
| Q2 | 93/4526 | 1.17(0.86, 1.57) | 0.315 | 1.11(0.81, 1.51) | 0.514 |
| Q3 | 140/4526 | 1.79(1.36, 2.35) | <0.001 | 1.59(1.17, 2.18) | 0.004 |
| Q4 | 194/4526 | 2.55(1.97, 3.31) | <0.001 | 1.98(1.36, 2.89) | <0.001 |
| *P* for trend |  |  | <0.001 |  | <0.001 |
| *P* for LRT |  |  | <0.001 |  | <0.001 |
| Model 1: unadjusted for covariates  Model 2: adjusted for age, sex, ethnicity (White and non-white), education (below high school level, high school and college or above), Townsend Deprivation Index, body mass index, smoking status (yes or no), excessive alcohol consumption (yes or no), HbA1c, hypertension (yes or no), use of statins (yes or no), FIB-4 (1.3< or ≥1.3)  Abbreviations: LRT, likelihood ratio tests. | | | | | |

**Supplementary Table 3. Associations between TyG-related indices and liver-related events in patients with type 2 diabetes after excluding participants who developed liver-related events within one year**

| **Characteristic** | **Event/ Number** | **Model 1** | | **Model 2** | |
| --- | --- | --- | --- | --- | --- |
|  |  | **HR (95% CI)** | **P value** | **HR (95% CI)** | **P value** |
| TyG-WC |  |  |  |  |  |
| Q1 | 70/4518 | Ref |  | Ref |  |
| Q2 | 78/4524 | 1.12 (0.81, 1.55) | 0.479 | 1.00 (0.71, 1.40) | 0.997 |
| Q3 | 148/4520 | 2.17 (1.63, 2.88) | <0.001 | 1.76 (1.27, 2.43) | <0.001 |
| Q4 | 190/4522 | 2.89 (2.20, 3.80) | <0.001 | 1.89 (1.28, 2.79) | 0.001 |
| P for trend |  |  | <0.001 |  | <0.001 |
| TyG-WHtR |  |  |  |  |  |
| Q1 | 71/4518 | Ref |  | Ref |  |
| Q2 | 89/4522 | 1.26 (0.92, 1.72) | 0.315 | 1.21 (0.87, 1.67) | 0.256 |
| Q3 | 135/4521 | 1.94 (1.46, 2.59) | <0.001 | 1.77 (1.27, 2.45) | <0.001 |
| Q4 | 191/4523 | 2.84 (2.16, 3.72) | <0.001 | 2.28 (1.55, 3.37) | <0.001 |
| P for trend |  |  | <0.001 |  | <0.001 |

Model 1: unadjusted for covariates

Model 2: adjusted for age, sex, ethnicity (White and others), education (below high school level, high school and college or above), Townsend Deprivation Index, body mass index, smoking status (never, former and current), excessive alcohol consumption (yes or no), HbA1c, hypertension (yes or no), use of statins (yes or no), FIB-4 (1.3<, 1.3-2.67, >2.67)

**Supplementary Table 4. Associations between the TyG-related indices and risk of liver-related events in patients with type 2 diabetes after additional adjusting for C-Reactive Protein**

| **Characteristic** | **Event/ Number** | **Model 1** | | **Model 2** | |
| --- | --- | --- | --- | --- | --- |
|  |  | **HR (95% CI)** | **P value** | **HR (95% CI)** | **P value** |
| TyG-WC |  |  |  |  |  |
| Q1 | 79/4527 | Ref |  | Ref |  |
| Q2 | 80/4526 | 1.02 (0.75, 1.39) | 0.897 | 0.91 (0.65, 1.25) | 0.500 |
| Q3 | 154/4526 | 1.99 (1.52, 2.62) | <0.001 | 1.58 (1.15, 2.16) | 0.004 |
| Q4 | 194/4526 | 2.61 (2.01, 3.39) | <0.001 | 1.63 (1.12, 2.39) | 0.011 |
| P for trend |  |  | <0.001 |  | <0.001 |
| TyG-WHtR |  |  |  |  |  |
| Q1 | 80/4527 | Ref |  | Ref |  |
| Q2 | 93/4526 | 1.17 (0.86, 1.57) | 0.315 | 1.12 (0.87, 1.53) | 0.500 |
| Q3 | 140/4526 | 1.79 (1.36, 2.35) | <0.001 | 1.63 (1.19, 2.23) | 0.002 |
| Q4 | 194/4526 | 2.55 (1.97, 3.31) | <0.001 | 2.00 (1.37, 2.91) | <0.001 |
| P for trend |  |  | <0.001 |  | <0.001 |

Model 1: unadjusted for covariates

Model 2: adjusted for age, sex, ethnicity (White and others), education (below high school level, high school and college or above), Townsend Deprivation Index, body mass index, smoking status (never, former and current), excessive alcohol consumption (yes or no), HbA1c, hypertension (yes or no), use of statins (yes or no), FIB-4 (1.3<, 1.3-2.67, >2.67), C-Reactive Protein

**Supplementary Table 5. Associations between TyG‑related indices and risk of incident liver‑related events in patients with type 2 diabetes after additional adjusting for** **dietary patterns**

| **Characteristic** | **Event/ Number** | **Model 1** | | **Model 2** | |
| --- | --- | --- | --- | --- | --- |
|  |  | **HR (95% CI)** | ***P* value** | **HR (95% CI)** | ***P* value** |
| TyG |  |  |  |  |  |
| Q1 | 28/1494 | Ref |  | Ref |  |
| Q2 | 30/1492 | 0.98 (0.58-1.66) | 0.948 | 0.95 (0.56-1.61) | 0.852 |
| Q3 | 37/1484 | 1.20 (0.72-2.00) | 0.480 | 1.18 (0.71-1.95) | 0.533 |
| Q4 | 30/1492 | 0.74 (0.42-1.31) | 0.298 | 0.72 (0.41-1.28) | 0.262 |
| *P* for trend |  |  | 0.441 |  | 0.402 |
| TyG-BMI |  |  |  |  |  |
| Q1 | 19/1503 | Ref |  | Ref |  |
| Q2 | 20/1502 | 0.96 (0.50-1.86) | 0.906 | 0.93 (0.48-1.81) | 0.836 |
| Q3 | 37/1484 | 1.62 (0.84-3.14) | 0.154 | 1.57 (0.81-3.05) | 0.182 |
| Q4 | 49/1473 | 1.82 (0.76-4.37) | 0.179 | 1.74 (0.73-4.18) | 0.212 |
| *P* for trend |  |  | 0.105 |  | 0.126 |
| TyG-WC |  |  |  |  |  |
| Q1 | 20/1502 | Ref |  | Ref |  |
| Q2 | 14/1508 | 0.64 (0.31-1.30) | 0.214 | 0.62 (0.31-1.27) | 0.192 |
| Q3 | 44/1477 | 1.78 (0.96-3.30) | 0.066 | 1.74 (0.94-3.21) | 0.079 |
| Q4 | 47/1475 | 1.37 (0.64-2.92) | 0.419 | 1.33 (0.62-2.84) | 0.457 |
| *P* for trend |  |  | 0.116 |  | 0.130 |
| TyG-WHtR |  |  |  |  |  |
| Q1 | 17/1505 | Ref |  | Ref |  |
| Q2 | 19/1503 | 1.19 (0.60-2.34) | 0.617 | 1.15 (0.58-2.26) | 0.684 |
| Q3 | 36/1485 | 2.00 (1.05-3.83) | 0.036 | 1.93 (1.01-3.69) | 0.047 |
| Q4 | 53/1469 | 2.91 (1.35-6.24) | 0.006 | 2.74 (1.28-5.89) | 0.010 |
| *P* for trend |  |  | 0.002 |  | 0.004 |

Model 1: adjusted for age, sex, ethnicity (White and non-white), education (below high school level, high school and college or above), Townsend Deprivation Index, body mass index, smoking status (yes or no), excessive alcohol consumption (yes or no), HbA1c, hypertension (yes or no), use of statins (yes or no), FIB-4 (1.3< or ≥1.3)

Model 2: further adjusted for the alternate Mediterranean diet (aMED) score

**Supplementary Table 6. Associations between TyG-related indices and liver-related events in patients with type 2 diabetes after** **imputation for missing covariates**

| **Characteristic** | **Event/ Number** | **Model 1** | | **Model 2** | |
| --- | --- | --- | --- | --- | --- |
|  |  | **HR (95% CI)** | **P value** | **HR (95% CI)** | **P value** |
| TyG-WC |  |  |  |  |  |
| Q1 | 86/5164 | Ref |  | Ref |  |
| Q2 | 89/5163 | 1.05 (0.78-1.41) | 0.771 | 0.93 (0.68-1.26) | 0.636 |
| Q3 | 171/5163 | 2.04 (1.57-2.64) | <0.001 | 1.64 (1.22-2.21) | 0.001 |
| Q4 | 216/5164 | 2.67 (2.08-3.43) | <0.001 | 1.77 (1.24-2.53) | 0.002 |
| P for trend |  |  | <0.001 |  | <0.001 |
| TyG-WHtR |  |  |  |  |  |
| Q1 | 85/5164 | Ref |  | Ref |  |
| Q2 | 112/5163 | 1.33 (1.00-1.76) | 0.050 | 1.25 (0.93-1.68) | 0.131 |
| Q3 | 151/5163 | 1.82 (1.39-2.37) | <0.001 | 1.64 (1.21-2.22) | 0.001 |
| Q4 | 214/5164 | 2.65 (2.06-3.41) | <0.001 | 2.09 (1.46-2.99) | <0.001 |
| P for trend |  |  | <0.001 |  | <0.001 |

Model 1: unadjusted for covariates

Model 2: adjusted for age, sex, ethnicity (White and others), education (below high school level, high school and college or above), Townsend Deprivation Index, body mass index, smoking status (never, former and current), excessive alcohol consumption (yes or no), HbA1c, hypertension (yes or no), use of statins (yes or no), FIB-4 (1.3<, 1.3-2.67, >2.67)

**Supplementary Table 7. Associations between TyG-related indices and liver-related events in patients with type 2 diabetes using competing risk models**

| **Characteristic** | **Event/ Number** | **Model 1** | | **Model 2** | |
| --- | --- | --- | --- | --- | --- |
|  |  | **HR (95% CI)** | **P value** | **HR (95% CI)** | **P value** |
| TyG-WC |  |  |  |  |  |
| Q1 | 79/4527 | Ref |  | Ref |  |
| Q2 | 80/4526 | 1.01 (0.74-1.38) | 0.950 | 0.91 (0.66-1.26) | 0.570 |
| Q3 | 154/4526 | 1.95 (1.49-2.56) | <0.001 | 1.58 (1.15-2.16) | 0.005 |
| Q4 | 194/4526 | 2.46 (1.89-3.20) | <0.001 | 1.58 (1.08-2.32) | 0.019 |
| P for trend |  |  | <0.001 |  | 0.002 |
| TyG-WHtR |  |  |  |  |  |
| Q1 | 80/4527 | Ref |  | Ref |  |
| Q2 | 93/4526 | 1.16 (0.86-1.57) | 0.330 | 1.12 (0.82-1.54) | 0.470 |
| Q3 | 140/4526 | 1.75 (1.33-2.30) | <0.001 | 1.57 (1.15-2.16) | 0.005 |
| Q4 | 194/4526 | 2.43 (1.87-3.15) | <0.001 | 1.89 (1.29-2.77) | 0.001 |
| P for trend |  |  | <0.001 |  | <0.001 |

Model 1: unadjusted for covariates

Model 2: adjusted for age, sex, ethnicity (White and others), education (below high school level, high school and college or above), Townsend Deprivation Index, body mass index, smoking status (never, former and current), excessive alcohol consumption (yes or no), HbA1c, hypertension (yes or no), use of statins (yes or no), FIB-4 (1.3<, 1.3-2.67, >2.67)

**Supplementary Table 8. Associations between TyG‑related indices and risk of incident liver‑related outcomes in patients with type 2 diabetes**

|  | **Cirrhosis** | | **Hepatocellular carcinoma** | | **Liver-related mortality** | |
| --- | --- | --- | --- | --- | --- | --- |
|  | **HR (95% CI)** | ***P* value** | **HR (95% CI)** | ***P* value** | **HR (95% CI)** | ***P* value** |
| TyG |  |  |  |  |  |  |
| Q1 | Ref |  | Ref |  | Ref |  |
| Q2 | 0.88 (0.67-1.16) | 0.375 | 0.91 (0.45-1.84) | 0.801 | 0.90 (0.49-1.66) | 0.730 |
| Q3 | 0.83 (0.63-1.10) | 0.193 | 1.93 (1.04-3.56) | 0.036 | 0.92 (0.50-1.70) | 0.797 |
| Q4 | 0.98 (0.74-1.30) | 0.887 | 1.63 (0.84-3.16) | 0.146 | 1.23 (0.68-2.25) | 0.492 |
| *P* for trend |  | 0.789 |  | 0.038 |  | 0.502 |
| TyG-BMI |  |  |  |  |  |  |
| Q1 | Ref |  | Ref |  | Ref |  |
| Q2 | 0.95 (0.69-1.32) | 0.777 | 1.55 (0.72-3.32) | 0.265 | 1.52 (0.69-3.34) | 0.303 |
| Q3 | 1.26 (0.89-1.77) | 0.192 | 2.83 (1.24-6.47) | 0.013 | 2.58 (1.13-5.89) | 0.024 |
| Q4 | 1.27 (0.80-2.03) | 0.314 | 5.07 (1.73-14.9) | 0.003 | 6.14 (2.21-17.0) | <0.001 |
| *P* for trend |  | 0.194 |  | 0.001 |  | <0.001 |
| TyG-WC |  |  |  |  |  |  |
| Q1 | Ref |  | Ref |  | Ref |  |
| Q2 | 0.89 (0.64-1.26) | 0.525 | 0.91 (0.41-2.02) | 0.810 | 1.10 (0.49-2.49) | 0.813 |
| Q3 | 1.59 (1.14-2.21) | 0.006 | 1.96 (0.93-4.13) | 0.078 | 2.18 (1.01-4.71) | 0.047 |
| Q4 | 1.63 (1.10-2.43) | 0.016 | 2.74 (1.14-6.59) | 0.025 | 2.82 (1.16-6.84) | 0.022 |
| *P* for trend |  | 0.001 |  | 0.008 |  | 0.008 |
| TyG-WHtR |  |  |  |  |  |  |
| Q1 | Ref |  | Ref |  | Ref |  |
| Q2 | 1.18 (0.85-1.64) | 0.335 | 1.06 (0.52-2.19) | 0.869 | 1.39 (0.67-2.89) | 0.376 |
| Q3 | 1.68 (1.20-2.34) | 0.002 | 1.79 (0.87-3.67) | 0.112 | 1.57 (0.73-3.37) | 0.247 |
| Q4 | 2.13 (1.43-3.17) | <0.001 | 2.15 (0.90-5.14) | 0.087 | 3.03 (1.29-7.12) | 0.011 |
| *P* for trend |  | <0.001 |  | 0.049 |  | 0.013 |

Model was adjusted for age, sex, ethnicity (White and non-white), education (below high school level, high school and college or above), Townsend Deprivation Index, body mass index, smoking status (yes or no), excessive alcohol consumption (yes or no), HbA1c, hypertension (yes or no), use of statins (yes or no), FIB-4 (1.3< or ≥1.3)

**Supplementary Table 9. Associations between TyG‑related indices and risk of incident liver‑related events in patients with type 2 diabetes (models without adjustment for BMI)**

| **Characteristic** | **Event/ Number** | **Model 1** | | **Model 2** | |
| --- | --- | --- | --- | --- | --- |
|  |  | **HR (95% CI)** | ***P* value** | **HR (95% CI)** | ***P* value** |
| TyG |  |  |  |  |  |
| Q1 | 118/4527 | Ref |  | Ref |  |
| Q2 | 115/4526 | 0.91 (0.70, 1.18) | 0.482 | 0.97 (0.75-1.25) | 0.799 |
| Q3 | 120/4526 | 0.92 (0.71, 1.19) | 0.526 | 0.98 (0.76-1.27) | 0.890 |
| Q4 | 154/4526 | 0.98 (0.75, 1.28) | 0.878 | 1.08 (0.82-1.41) | 0.593 |
| *P* for trend |  |  | 0.901 |  | 0.592 |
| TyG-BMI |  |  |  |  |  |
| Q1 | 86/4527 | Ref |  | Ref |  |
| Q2 | 96/4526 | 1.00 (0.74, 1.37) | 0.989 | 1.08 (0.81-1.45) | 0.604 |
| Q3 | 144/4526 | 1.31 (0.94, 1.81) | 0.111 | 1.50 (1.14-1.98) | 0.003 |
| Q4 | 181/4526 | 1.44 (0.92, 2.24) | 0.106 | 1.92 (1.46-2.53) | <0.001 |
| *P* for trend |  |  | <0.001 |  | <0.001 |
| TyG-WC |  |  |  |  |  |
| Q1 | 79/4527 | Ref |  | Ref |  |
| Q2 | 80/4526 | 0.90(0.65, 1.24) | 0.509 | 0.96 (0.70-1.31) | 0.795 |
| Q3 | 154/4526 | 1.57(1.15, 2.15) | 0.005 | 1.77 (1.34-2.35) | <0.001 |
| Q4 | 194/4526 | 1.63(1.12, 2.38) | 0.012 | 2.02 (1.52-2.69) | <0.001 |
| *P* for trend |  |  | <0.001 |  | <0.001 |
| TyG-WHtR |  |  |  |  |  |
| Q1 | 80/4527 | Ref |  | Ref |  |
| Q2 | 93/4526 | 1.11(0.81, 1.51) | 0.514 | 1.15 (0.85-1.55) | 0.370 |
| Q3 | 140/4526 | 1.59(1.17, 2.18) | 0.004 | 1.71 (1.29-2.26) | <0.001 |
| Q4 | 194/4526 | 1.98(1.36, 2.89) | <0.001 | 2.24 (1.69-2.97) | <0.001 |
| *P* for trend |  |  | <0.001 |  | <0.001 |

Model 1: adjusted for age, sex, ethnicity (White and non-white), education (below high school level, high school and college or above), Townsend Deprivation Index, body mass index, smoking status (yes or no), excessive alcohol consumption (yes or no), HbA1c, hypertension (yes or no), use of statins (yes or no), FIB-4 (1.3< or ≥1.3)

Model 2: model 1 without adjustment for body mass index


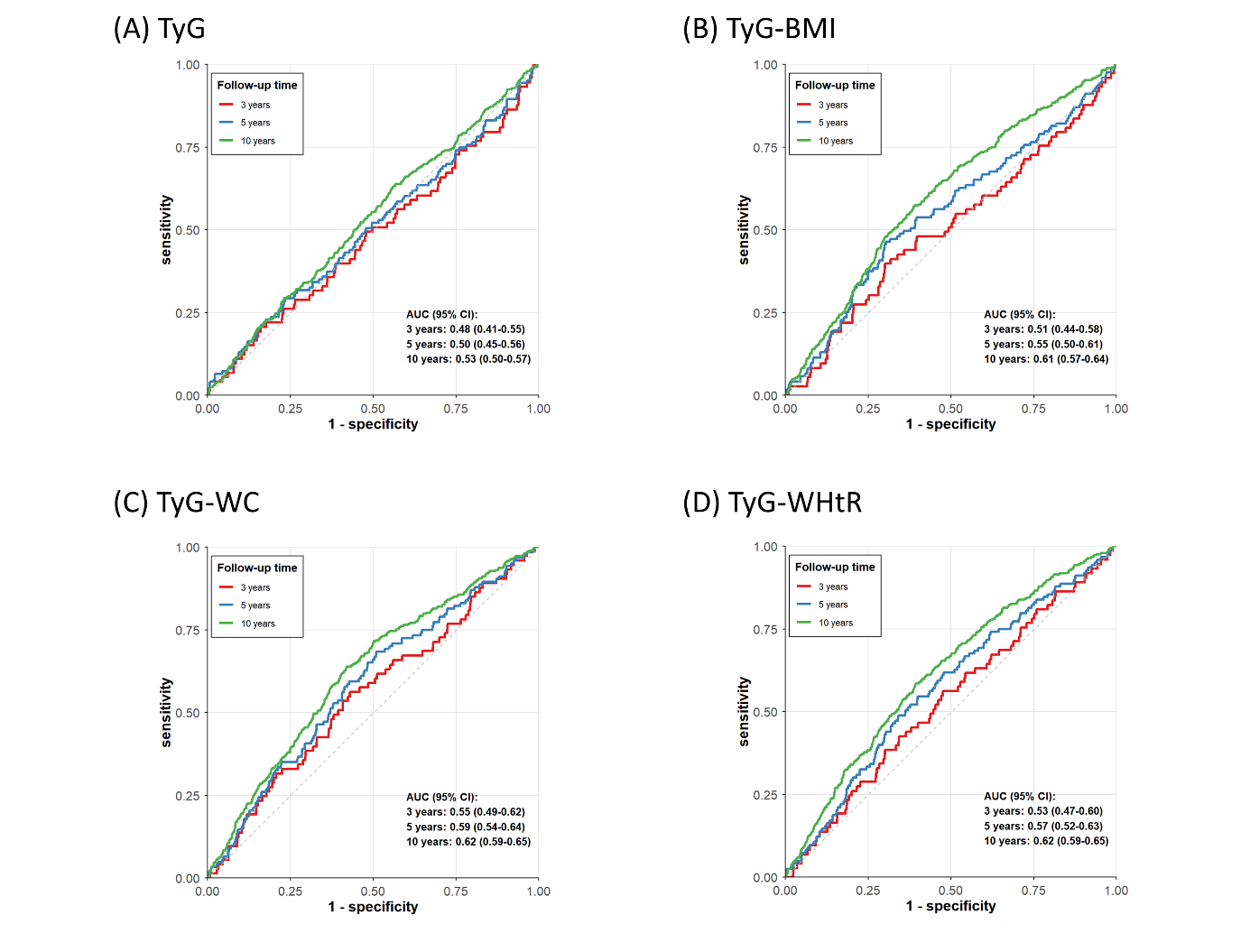
 Supplementary Figure 1. Time‑dependent ROC curves of TyG‑related indices for predicting liver-related events in patients with type 2 diabetes.
